# Gut Commensal Bacteria Ameliorate Liver Fibrosis by Inhibiting Macrophage M1 Polarization Through Secondary Bile Acids in a Murine Liver Fibrosis Model

**DOI:** 10.3390/microorganisms14092035

**Published:** 2026-09-12

**Authors:** Hongyan Xiang, Huanyu Xiang, Zongyi Liu, Hang Chen, Zhidan Luo, Shuliang Guo, Jie Zhang

**Affiliations:** 1 Department of Geriatrics, Chongqing Academy of Medical Sciences, Chongqing General Hospital, Chongqing University, Chongqing 401147, China; xianghy_30164424@cqu.edu.cn (H.X.);; 2 The Key Laboratory of Molecular Biology for Infectious Diseases, Department of Infectious Diseases, Institute for Viral Hepatitis, The Second Affiliated Hospital of Chongqing Medical University, Chinese Ministry of Education, Chongqing 400010, China; 3 Institute of Hepatopancreatobiliary Surgery, Chongqing Academy of Medical Sciences, Chongqing General Hospital, Chongqing University, Chongqing 401147, China; 4 Department of Respiratory and Critical Care Medicine, The First Affiliated Hospital of Chongqing Medical University, Chongqing 400016, China

**Keywords:** gut microbiota, bile acids, liver fibrosis, metabolic reprogramming of macrophages, gut–liver axis

## Abstract

The gut microbiota plays a critical role in regulating liver metabolism and immunity, significantly influencing hepatic inflammation and fibrogenesis. However, the mechanisms by which it modulates the progression of liver fibrosis remain unclear. This study investigated the interplay between the gut microbiota and bile acids in murine liver fibrosis. A mouse model of liver fibrosis was established via intraperitoneal injection of carbon tetrachloride. The gut microbiota was depleted using an antibiotic cocktail, as confirmed through 16S rRNA sequencing. Bile acid profiles were measured using ultra-performance liquid chromatography–tandem mass spectrometry. The effects of secondary bile acids on macrophage polarization were assessed in vivo and in vitro. Gut commensal microbiota depletion exacerbated liver inflammation and fibrosis. Concomitantly, this depletion significantly reduced microbiota-derived secondary bile acids. Notably, supplementation with deoxycholic acid markedly attenuated liver fibrosis. This protective effect was associated with the inhibition of pro-inflammatory M1 macrophage polarization. Takeda G protein-coupled receptor 5 (TGR5) activation was identified as the mechanism underlying the effect of deoxycholic acid on macrophages, as a TGR5 antagonist reversed this inhibition. Mechanistically, TGR5 activation suppressed the NF-κB pathway in macrophages via the cAMP–PKA signaling cascade, thereby inhibiting hepatic stellate cell activation. Our results demonstrate that the gut microbiota–secondary bile acid–TGR5 signaling axis plays a critical role in liver fibrosis and represents a promising therapeutic target.

## 1. Introduction

Liver fibrosis, marked by an overabundance of extracellular matrix (ECM) deposition, is a pathological endpoint shared by all chronic liver diseases (CLDs). The progression of fibrosis ultimately leads to cirrhosis and subsequent hepatocellular carcinoma (HCC), which collectively impose a substantial global disease burden and are major causes of liver-related mortality [1]. Persistent and excessive hepatic inflammation, triggered by repetitive or chronic hepatocyte injury, regardless of the etiology, is a central driver of the occurrence and progression of liver fibrosis. Beyond primary liver injury, emerging evidence has highlighted the gut microbiota as a key modulator of liver metabolism and immune responses, significantly influencing both hepatic inflammation and fibrogenesis [2,3,4].

The special anatomical and functional connection between the liver and intestine enables the gut microbiota to impact liver inflammation and fibrogenesis. Specifically, the liver is constantly exposed to gut microbes and metabolites through the portal vein. Both clinical and experimental studies have found notable shifts in microbiota composition, referred to as dysbiosis, that are associated with fibrotic liver disease [5]. Crucially, this dysbiosis critically affects the generation of microbial metabolites, which serve as vital mediators of the effects that the gut microbiota exerts on the host and are strongly implicated in various inflammation-driven pathologies [6,7]. Despite these advances, the precise mechanisms by which gut-derived microbes and their metabolites drive hepatic fibrogenesis remain unclear and require further investigation.

Bile acids (BAs), which are synthesized in the liver and modified by gut bacteria, are crucial signaling molecules that mediate gut–liver crosstalk. Dysregulation of BA signaling is particularly relevant in CLDs, because disruptions of this crosstalk can interfere with the transcriptional networks governing inflammation, fibrosis, and endothelial dysfunction [8], thereby driving the development of nonalcoholic fatty liver disease [9], cholestatic injury [10], and HCC [11]. Studies utilizing clinical specimens have identified distinct BA species as sensitive biomarkers of fibrogenesis or cirrhosis, revealing their diagnostic potential [12,13,14,15]. Preclinical evidence further substantiates that both BAs and their synthetic analogs attenuate hepatic fibrosis through targeted activation of cognate receptor signaling pathways, revealing their therapeutic potential in combating fibrotic remodeling [16,17]. These converging lines of evidence definitively establish BA metabolism as a central node connecting gut microbiota dynamics with hepatic fibrotic remodeling, underscoring the necessity to elucidate the precise microbial–BA crosstalk governing this process.

In the present study, we found that the commensal microbiota confers protection against hepatic inflammation and fibrogenesis, with microbial-derived secondary BAs functioning as central orchestrators of this process. Mechanistically, secondary BAs mitigate liver injury-associated inflammation through targeted activation of the Takeda G protein-coupled receptor 5 (TGR5), effectively suppressing macrophage polarization toward pro-inflammatory phenotypes. Importantly, these findings position the gut microbiota–BA axis as an innovative therapeutic target for future anti-fibrotic strategies.

## 2. Materials and Methods

### 2.1. Mice

Specific pathogen-free male C57BL/6J mice (Vital River, Beijing, China), aged 6–7 weeks with body weights of 18–22 g, were conditioned in climate-controlled isolators. Environmental settings were maintained at 24 °C ± 1 °C and 50% ± 5% relative humidity. Animals had access to autoclaved water and standard chow ad libitum under 12 h diurnal cycles. Following a 7-day acclimatization period, the subjects were randomly assigned to experimental cohorts. All animal protocols followed the ARRIVE guidelines, were approved by the Laboratory Animal Welfare and Ethics Committee, and adhered to the Animal Ethics Statement of the Ethics Committee of the Second Affiliated Hospital of Chongqing Medical University.

### 2.2. Gut Microbiota Depletion Protocol

Animals underwent daily oral administration of a non-absorbable broad-spectrum antibiotic cocktail (ABX: metronidazole 1 mg/mL, ampicillin 1 mg/mL, neomycin sulfate 1 mg/mL, and vancomycin 0.5 mg/mL in sterile phosphate-buffered saline [PBS]) via 200 μL gavage for seven consecutive days (Sangon Biotech, Shanghai, China). During the subsequent 6-week fibrotic induction period, ABX maintenance was achieved through ad libitum access to antibiotic-supplemented water, with the solution refreshed every 48 h to ensure potency.

### 2.3. Liver Fibrosis Model

To establish a mouse liver fibrosis model, carbon tetrachloride (CCl_4_; 20% *v*/*v* in olive oil) was intraperitoneally administered to mice at 5 mL/kg twice weekly for six consecutive weeks. Control cohorts received equivalent volumes of the olive oil vehicle following identical administration protocols.

### 2.4. Deoxycholic Acid Intervention

The experimental subjects received 30 mg/kg deoxycholic acid (DCA) (264101; Sigma-Aldrich, St. Louis, MO, USA) dissolved in PBS via oral gavage 1 h prior to each CCl_4_ challenge [18]. Vehicle control groups received PBS following an identical administration protocol.

### 2.5. 16S rRNA Gene Sequencing and Bioinformatic Analysis

Fecal microbial DNA was isolated using a Microbiome DNA Kit (MJYH, Shanghai, China), according to the manufacturer’s specifications. The bacterial 16S rRNA V3-V4 hypervariable regions were subsequently PCR-amplified, gel-purified, and subjected to paired-end sequencing following published methodologies [19]. Raw sequencing reads underwent rigorous quality control to generate high-fidelity consensus sequences [19,20], followed by denoising with DADA2 for the precise resolution of amplicon sequence variants (ASVs). All subsequent analyses leveraged this ASV-defined microbiome profile. Bioinformatic analyses were conducted on the Majorbio Cloud Platform (https://cloud.majorbio.com).

### 2.6. Aerobic Cultivation of Gut Bacteria

To evaluate gut microbiota depletion following ABX treatment, cecal contents were aseptically harvested from control and ABX-treated mice at experimental termination. Fresh cecal specimens were weighed and transferred into 1.5 mL sterile Eppendorf tubes containing 1 mL of sterile PBS, followed by vortexing for 5 min at room temperature. Serial dilutions were performed until reaching 10^−4^, and sample homogenization was ensured by vortexing for 15 s before removing the respective volume. Aliquots (100 μL) from the undiluted suspension and serial dilutions (10^−3^ and 10^−4^) were streaked onto lysogeny broth agar plates (Solarbio Life Sciences, Beijing, China). Bacterial cultures were aerobically maintained at 37 °C for 24 h in humidity-regulated chambers. Viable counts were calculated and standardized to colony-forming units per gram.

### 2.7. Alanine and Aspartate Aminotransferase Measurements

Serum alanine aminotransferase (ALT) and aspartate aminotransferase (AST) levels were measured using commercial enzymatic assay kits in strict adherence to the manufacturer’s operating procedures.

### 2.8. Histological Analysis

Following euthanasia via intraperitoneal injection of 1% pentobarbital sodium, representative mouse liver sections were harvested for histopathological examination. Hematoxylin–eosin staining was used to observe inflammation, and Sirius Red (SR) staining was used to detect fibrosis, according to standard procedures. Immunohistochemical profiling of collagen I and α-SMA was performed according to an established methodology [21]. Briefly, overnight incubation at 4 °C with primary antibodies (anti-collagen I: ab270993, 1:500, Abcam, Cambridge, UK; anti-α-SMA: 14395-1-AP, 1:4000, Proteintech, Rosemont, IL, USA) preceded the application of a goat anti-rabbit secondary antibody (GB23303, 1:500, Servicebio, Wuhan, China). Chromogenic development was performed using diaminobenzidine with hematoxylin counterstaining, followed by dehydration.

For immunofluorescence staining, the tissue sections were deparaffinized, rehydrated, antigen-retrieved, and blocked, as described above. The sections were incubated with an anti-F4/80 antibody (GB11027; 1:500, Servicebio), followed by incubation with a secondary antibody (GB21303; 1:100, Servicebio). For cellular fluorescence staining, fixed cells were incubated with an anti-CD86 primary antibody (13395-1-AP; 1:100, Proteintech), followed by incubation with a secondary antibody (SA00013-2; 1:100, Proteintech). Finally, 4′,6-diamidino-2-phenylindole was used to counterstain the nuclei.

Whole-slide scanning was performed on the stained tissue sections using a Pannoramic DESK scanner (3DHistech, Budapest, Hungary). The digital slides were then quantitatively analyzed in a blinded manner by two senior pathologists using ImageJ v.1.52 (National Institutes of Health, Bethesda, MD, USA).

### 2.9. Total RNA Extraction and Quantitative RT-PCR

Total RNA was isolated from liquid nitrogen-preserved tissues or cell pellets using TRIzol reagent (Invitrogen, Waltham, MA, USA). For cDNA preparation, aliquots of 1000 ng of total RNA (DNase-treated) were reverse-transcribed with PrimeScript RT Master Mix incorporating gDNA elimination technology (Takara, Kusatsu, Japan). The cDNA was amplified via real-time PCR reactions with EnzyArtisan Universal SYBR Green Master Mix (Shanghai, China) and gene-specific primers (Supplementary Table S1).

### 2.10. Western Blotting

Total protein was extracted using a commercial lysis assay (KeyGen Biotech, Nanjing, China), followed by protein quantitation using a BCA kit (KeyGen Biotech). Immunoblotting was performed using the following primary antibodies at the specified dilutions: anti-α-SMA (1:1000; Proteintech), anti-collagen I (1:1000; Proteintech), anti-iNOS (1:1000; Proteintech), anti-TNF-α (1:500; Affinity Biosciences, Cincinnati, OH, USA), anti-CD86 (1:1000; Affinity Biosciences), anti-p-p65 (1:1000; Abcam), anti-p65 (1:5000; Abcam), anti-p-IκB (1:1000; Cell Signaling Technology, Danvers, MA, USA), anti-IκB (1:1000; Cell Signaling Technology), anti-β-actin (1:10,000; Proteintech), and anti-TGR5 (1:1000; Abcam). Following primary antibody incubation, the membranes were probed with horseradish peroxidase-conjugated secondary antibodies. Protein bands were digitally captured (BioSpectrum Gel Imaging System) and quantified using ImageJ with β-actin normalization.

### 2.11. BA Analysis

BAs present in distal ileal contents were analyzed using an ultra-performance liquid chromatography–tandem mass spectrometry platform by Metabo-Profile Inc. (Shanghai, China), as previously described, with several modifications [22]. Briefly, ileal contents (10 mg) underwent mechanical lysis with 25 mg of cryo-cooled grinding beads in 200 μL of an acetonitrile/methanol solution (4:1) containing 10 μL of internal standard. Following homogenization, the samples were centrifuged (18,000× *g*, 20 min, 4 °C) to pellet debris, and the supernatants were retained for subsequent analysis. The supernatants, clarified through vortex–centrifugation, were cryo-archived at −80 °C pending LC-MS analysis. BA quantitation was achieved using QuanMET v.2.0 for peak integration, calibration curve fitting, and analyte quantification. Data are reported as nmol BA per gram of ileal content (nmol·g^−1^).

### 2.12. Flow Cytometry Analysis

Intrahepatic mononuclear cells were isolated following established protocols with modifications [23,24]. Briefly, fresh mouse livers were mechanically dispersed through 200-mesh screens in PBS, followed by centrifugation at 50× *g* for 5 min to obtain the cell supernatant. The supernatant was then centrifuged at 413× *g* for 10 min, and the resulting cell pellet was collected. The cell pellet was resuspended in 40% Percoll (Cytiva, Marlborough, MA, USA) and centrifuged at 860× *g* for 15 min. After the lysis of red blood cells, mononuclear cells were collected for subsequent flow cytometric analysis.

Fc receptor blockade was achieved through a 5 min pre-incubation with anti-CD16/CD32 (156603, 0.25 µg/10^6^ cells in 100 μL). Next, fluorescent anti-mouse CD11b-FITC (101205), F4/80-PE (123109), CD45-PerCP/Cyanine5.5 (103131), CD206-PC (141707), and CD86-Brilliant Violet (105031) antibodies (all sourced from BioLegend, San Diego, CA, USA, 0.25 µg/10^6^ cells in 50 μL) were used to identify macrophages and their subsets. Flow cytometry was performed on a Beckman Coulter CytoFLEX platform (Beckman Coulter, Brea, CA, USA).

### 2.13. Cell Culture and Treatment

The human hepatic stellate cell (HSC) line, LX-2, and human monocytic leukemia cell line, THP-1, were cultured in Roswell Park Memorial Institute (RPMI)-1640 medium (BioChannel, Nanjing, China) supplemented with 10% fetal bovine serum (FBS; BioChannel) and 1% penicillin–streptomycin (P/S; Gibco, Grand Island, NY, USA). RAW264.7 cells were cultured in Dulbecco’s Modified Eagle Medium (Gibco) supplemented with 10% FBS and 1% P/S. All cell lines were subjected to STR authentication.

Bone marrow-derived macrophages (BMDMs) were isolated from the tibiae and femurs of 8-week-old male C57BL/6J mice [25]. Cells underwent 7-day differentiation in complete medium supplemented with 10 ng/mL of recombinant mouse M-CSF (PeproTech, Cranbury, NJ, USA), with the medium refreshed on days 3 and 5. Terminally differentiated macrophages were collected on day 7 for subsequent assays.

To induce pro-inflammatory macrophage activation, RAW264.7 cells, BMDMs, and THP-1-derived macrophages were stimulated with 100 ng/mL of lipopolysaccharide (LPS; Sigma-Aldrich) for 24 h. For DCA treatment, the cells were pre-incubated with DCA at final concentrations of 25, 50, 75, or 100 μM for 3 h prior to LPS stimulation. The DCA concentrations were based on a published protocol, with modifications [26,27]. For inhibitor studies, cells were pretreated with SBI-115 (20 μM), H89 (10 μM), or MDL12330A (10 μM) for 1 h before DCA (50 μM) addition.

To determine the indirect actions of DCA on LX-2 activation, a THP-1/LX-2 co-culture platform was employed. THP-1 monocytes underwent M0 polarization after 48 h of exposure to 100 ng/mL of phorbol 12-myristate 13-acetate (MedChemExpress, Monmouth Junction, NJ, USA) followed by a 24 h resting period. The resulting M0 macrophages were polarized to the M1 phenotype with 100 ng/mL of LPS, preconditioned with or without 50 μM DCA. After washing with PBS, the cells were maintained in fresh complete medium for 24 h. Conditioned media (CM) were prepared through centrifugation (103× *g*, 10 min, 4 °C) of the culture supernatants, followed by cryopreservation at −80 °C. For co-culture experiments, LX-2 cells were inoculated into 6-well plates at 3 × 10^5^ cells/well. Following 24 h of adherence, the cells were exposed to 1:1 mixtures of CM and fresh RPMI-1640 medium from the specified THP-1 groups for 24 h.

To evaluate the direct effect of DCA on LX-2 cell activation, LX-2 cells were treated with 10 ng/mL of TGF-β1 (MedChemExpress) alone or in combination with varying concentrations of DCA (25, 50, 75, and 100 μM) for 24 h. After treatment, the cells were collected for gene and protein expression analyses.

### 2.14. Statistical Analysis

Statistical processing was performed using IBM SPSS Statistics v.26.0 (IBM, Armonk, NY, USA), with graphical rendering conducted in GraphPad Prism v.9.0.0 (GraphPad Software, San Diego, CA, USA). Values denote the mean ± standard error of the mean, unless annotated otherwise. For two-group comparisons, statistical significance was determined using Student’s *t*-test (parametric) or the Mann–Whitney *U* test (non-parametric). For multigroup comparisons, one-way ANOVA (parametric) or Kruskal–Wallis tests (non-parametric) were applied, followed by Tukey’s post hoc tests (for ANOVA) or Dunn’s tests (for Kruskal–Wallis). All tests were two-tailed, and statistical significance was defined as *p* < 0.05. Additional analytical details are provided in the relevant figure legends.

## 3. Results

### 3.1. Gut Microbiota Depletion Exacerbates Liver Injury and Fibrosis

Evidence suggests that the gut commensal microbiota confers protection against liver fibrosis [28]; however, the mechanisms underlying this effect remain unclear. To investigate the functional roles and mechanisms of the gut microbiota in fibrogenesis, we used ABX-treated mice to deplete commensal bacteria (Figure S1A). The efficacy of ABX treatment was confirmed through cecal content culture and fecal 16S rRNA gene sequencing. ABX had no detrimental impact on general health or body weight but markedly reduced the gut microbiota load, as quantified using aerobic culture (Figure S1B). Accordingly, ABX treatment significantly reduced microbial richness (Ace index) and diversity (Shannon index) in the 16S rRNA sequencing data (Figure S1C,D). Meanwhile, treatment with ABX resulted in a pronounced alteration in microbiota composition, as was evident from the distinct clustering observed in the Principal Coordinate Analysis based on Bray–Curtis dissimilarity (Figure S1E). Taxonomic analysis showed a shift from Bacteroidales/Lactobacillales dominance in conventional fibrotic mice to Enterobacteriales dominance in ABX-treated mice (Figure S1F). These findings indicate that ABX successfully disrupted and eliminated most of the murine gut microflora.

**Figure 1:**
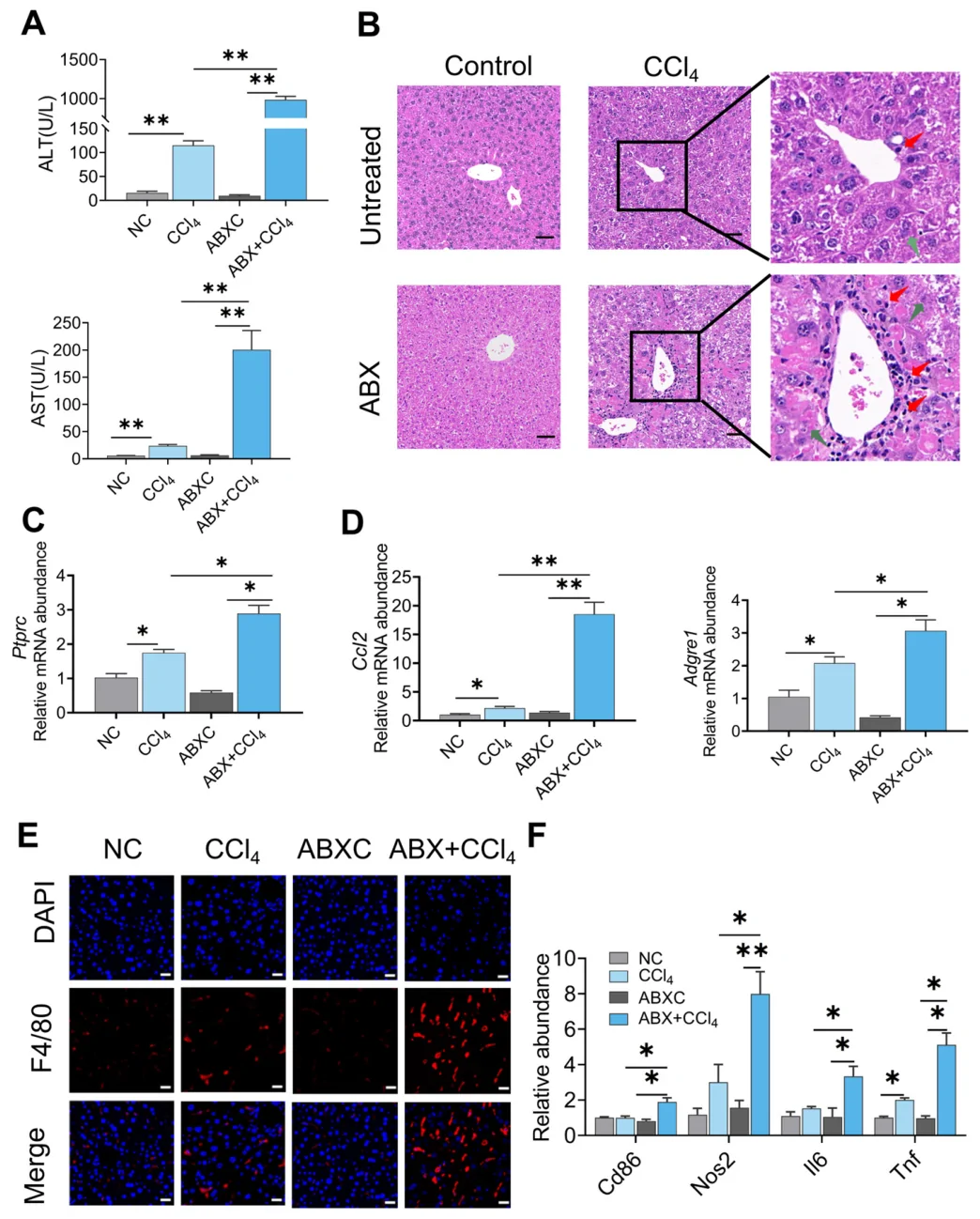
Gut microbiota depletion exacerbates CCl_4_-induced liver inflammation and injury. (**A**) Serum ALT and AST levels (n = 5–6). (**B**) Representative liver sections stained with H&E (original magnification ×200; scale bar: 50 μm). Inflammatory cells (red arrows); hepatocyte necrosis (green arrows). (**C**,**D**) Relative abundance of Ptprc (Cd45), Ccl2 (Mcp1), and Adgre1 (F4/80) mRNA levels in liver tissue (n = 4). (**E**) Representative immunofluorescence staining of F4/80 (original magnification ×400, scale bar: 25 μm). (**F**) Relative mRNA expression of M1-macrophage markers Cd86, Nos2, Il6, and Tnf (n = 4). Comparisons between two groups were performed using two-tailed Student’s *t*-tests or Mann–Whitney *U* tests, as appropriate. The results are presented as mean ± SEM. * *p* < 0.05, ** *p* < 0.01. NC, normal control; CCl_4_, carbon tetrachloride; ABX, broad-spectrum antibiotic cocktail; ABXC, ABX control.

ABX-treated mice exhibited significantly elevated serum ALT and AST levels relative to those of the controls following repeated CCl_4_ challenges (Figure 1A). Similarly, relative to the untreated fibrotic mice, the ABX-treated group exhibited exacerbated hepatic pathology, including inflammatory cell infiltration, hepatocyte swelling, and necrosis (Figure 1B). Furthermore, the ABX-treated fibrosis group exhibited enhanced leukocyte infiltration, as evidenced by their significantly elevated *Ptprc* (Cd45) mRNA expression compared to that of the untreated fibrotic mice (Figure 1C). The RT-qPCR and immunofluorescence results further confirmed that macrophages were the predominant infiltrating cell type (Figure 1D,E), with a particular increase in pro-inflammatory M1 macrophages (Figure 1F).

**Figure 2:**
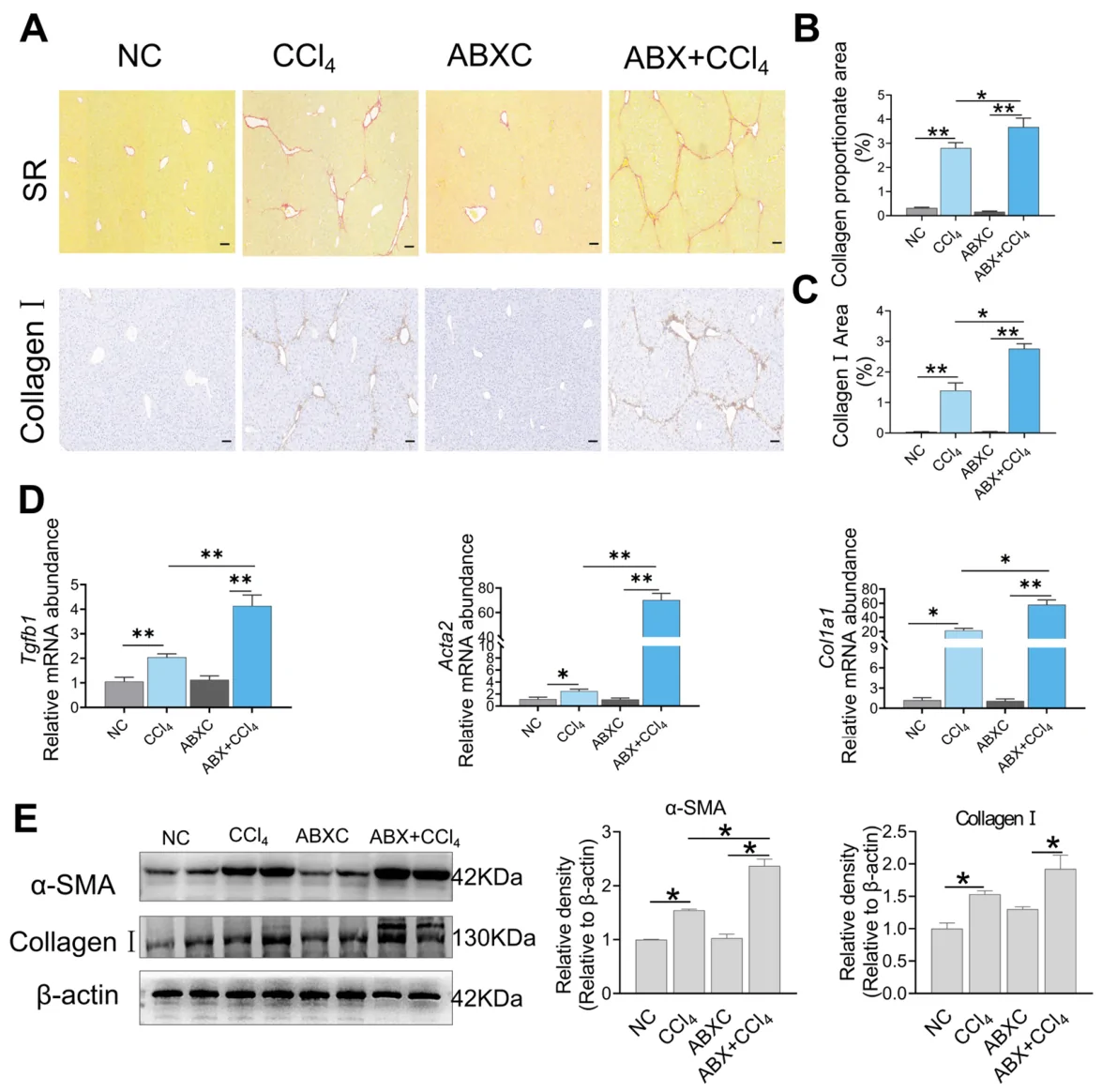
Gut microbiota depletion exacerbates CCl_4_-induced liver fibrosis. (**A**) Representative liver sections stained with Sirius Red (SR; collagen deposition) and immunohistochemistry (IHC) for collagen I (original magnification ×100; scale bar: 100 μm). (**B**) Semi-quantitation of SR staining–positive area by ImageJ (n = 6). (**C**) Semi-quantitative analysis of collagen I (n = 3). (**D**) Relative mRNA expression of fibrosis-related genes in liver tissue (n = 4). (**E**) Hepatic protein expression of α-SMA and collagen I by immunoblotting (n = 4). Comparisons between two groups were performed using two-tailed Student’s *t*-tests or Mann–Whitney *U* tests, as appropriate. The results are presented as mean ± SEM. * *p* < 0.05, ** *p* < 0.01. NC, normal control; CCl_4_, carbon tetrachloride; ABX, broad-spectrum antibiotic cocktail; ABXC, ABX control.

Collagen deposition was quantified via SR staining to assess the fibrosis caused by chronic liver injury. Marked collagen accumulation was observed in CCl_4_-treated mice versus control mice, with further augmentation following ABX administration (Figure 2A,B). The ECM product, collagen I, was also significantly elevated in ABX-treated mice compared to that in untreated fibrotic mice (Figure 2A,C). The expression levels of HSC activation-related genes, including actin alpha 2 smooth muscle (*Acta2*), transforming growth factor beta (*Tgfb*), and collagen type I alpha 1 chain (*Col1a1*), were significantly higher in the antibiotic-treated group compared to those in the control mice following CCl_4_ administration (Figure 2D). Consistent with the upregulated mRNA levels, HSC activation markers (α-SMA, collagen I) were elevated in CCl_4_-treated mice and further increased by ABX co-treatment (Figure 2E). Collectively, these data demonstrate that gut microbiota depletion aggravates inflammatory damage and fibrosis in CCl_4_-induced liver injury.

### 3.2. Gut Microbiota Depletion Reduces Secondary BA Levels

**Figure 3:**
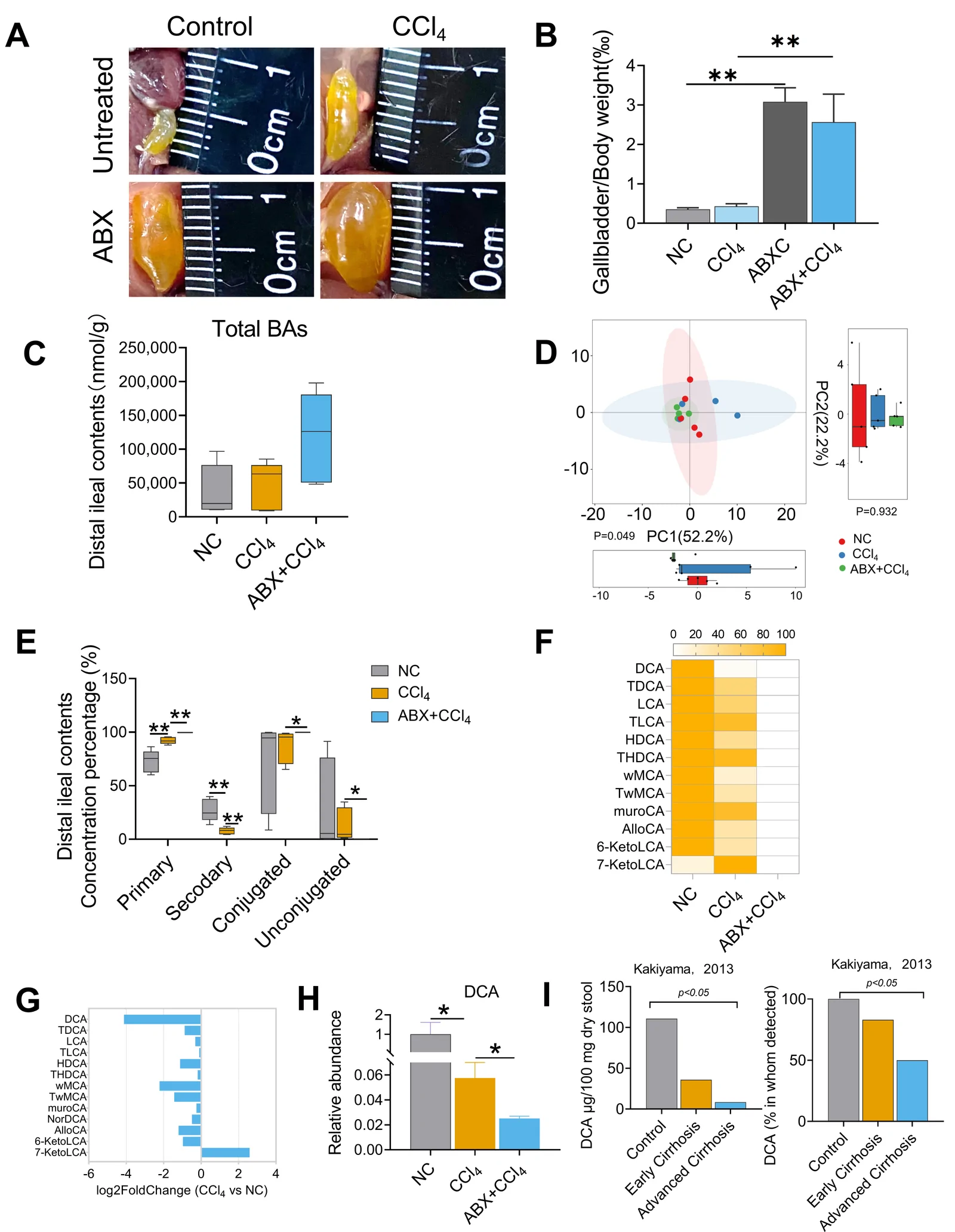
Gut microbiota depletion by ABX disrupts BA metabolism. (**A**) Gallbladder size. (**B**) Gallbladder-to-liver mass ratio (n = 6). The results are presented as mean ± SEM. (**C**) Total bile acid (BA) levels in the distal ileum (n = 5). Box plots show the median ± interquartile range (IQR) and 1.5 IQR of the quartiles (whiskers). (**D**) Principal component analysis (PCA) of BA composition in distal ileal content across groups. (**E**) Levels of primary, secondary, conjugated, and unconjugated BA in distal ileum (n = 4–5). (**F**) Heatmap of secondary BA concentrations (scaled 0–100) across experimental groups. (**G**) The fold change of secondary BAs in the CCl_4_ group compared to the normal control group. (**H**) DCA levels in different groups (n = 4–5). (**I**) Fecal DCA distribution in control, early cirrhosis, and advanced cirrhosis patients (data from indicated literature). Mann–Whitney *U* tests were used for BA analysis between two groups. * *p* < 0.05, ** *p* < 0.01. NC, normal control; CCl_4_, carbon tetrachloride; ABX, broad-spectrum antibiotic cocktail; ABXC, ABX control; DCA, deoxycholic acid; TDCA, taurodeoxycholic acid; LCA, lithocholic acid; TLCA, taurolithocholic acid; HDCA, hyodeoxycholic acid; THDCA, taurohyodeoxycholic acid; wMCA, omega-muricholic acid; TwMCA, tauro-omega-muricholic acid; muroCA, murocholic acid; NorDCA, nor-deoxycholic acid; AlloCA, allocholic acid; 6-KetoLCA, 6-ketolithocholic acid; 7-KetoLCA, 7-ketolithocholic acid [30].

Our previous study established dysregulated BA metabolism in rat liver fibrosis [29]. Building upon this foundation, the current study demonstrated that ABX-treated fibrotic mice exhibited exacerbated BA dysregulation compared to that in untreated fibrotic controls. This aggravation was directly indicated by the ABX-induced enlargement of the gallbladder BA pool (Figure 3A,B) and elevated total BA levels in the distal ileum (Figure 3C). In addition, the BA composition was significantly altered in response to CCl_4_ induction and further modified by ABX treatment (Figure 3D). Specifically, compared to the control mice, CCl_4_-induced liver fibrosis resulted in increased primary BA levels and decreased secondary BA levels. This shift in BA composition was amplified by ABX treatment in the fibrotic mice (Figure 3E).

A decline in secondary BA levels is implicated in fibrosis progression across nonalcoholic steatohepatitis [31], hepatitis B virus infection [15], and primary biliary cirrhosis [32]. Our data revealed consistent reductions in most individual secondary BAs during hepatic fibrogenesis, with DCA exhibiting the most striking decline (Figure 3F–H). Notably, this reduction was significantly exacerbated in microbiota-depleted mice compared to that in controls following CCl_4_ exposure, consistent with the established role of the gut microbiota in regulating secondary BA synthesis. Analysis of public datasets further revealed a progressive decline in secondary BAs from healthy controls to patients with early and advanced cirrhosis, irrespective of etiology. Moreover, both the concentration and detection frequency of DCA progressively decreased with cirrhosis severity (Figure 3I) [30]. Collectively, these findings suggest that secondary BAs, particularly DCA, may function as key microbial-derived mediators that modulate the progression of liver fibrosis.

### 3.3. Microbiota-Derived DCA Alleviates Liver Fibrosis

**Figure 4:**
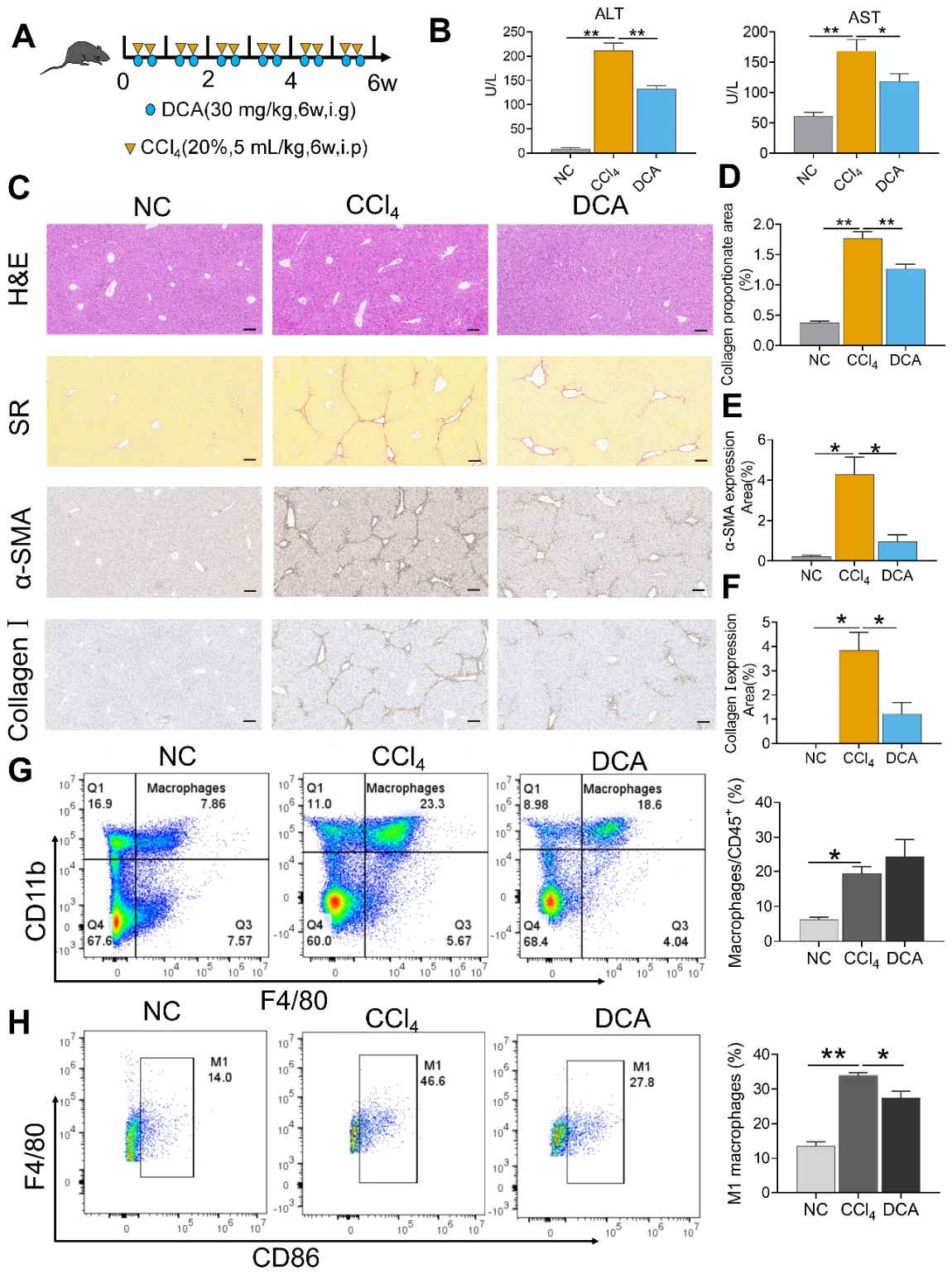
DCA alleviates liver fibrosis in CCl_4_-induced liver fibrosis. (**A**) Experimental design schematic. (**B**) Serum levels of ALT and AST (n = 5–7). (**C**) Representative images of liver specimens stained with H&E, SR, and IHC analyses of α-SMA and collagen I (original magnification ×100; scale bar: 100 μm). Semi-quantitative analysis of (**D**) Sirius Red (SR)-positive area (n = 5–7), (**E**) α-SMA–positive area (n = 3), and (**F**) collagen I–positive area (n = 3) was conducted using ImageJ software. (**G**,**H**) Flow cytometry analysis of the percentage of macrophages and M1 macrophages in the liver of representative mice in the NC, CCl_4_, and DCA treatment groups (n = 3). Comparisons between two groups were performed using two-tailed Student’s *t*-tests or Mann–Whitney *U* tests, as appropriate. The results are presented as mean ± SEM. * *p* < 0.05, ** *p* < 0.01. NC, normal control; CCl_4_, carbon tetrachloride; DCA, deoxycholic acid; mice treated with DCA and CCl_4_.

DCA was selected for mechanistic validation to elucidate the contribution of secondary BAs to hepatic fibrosis. Mice received oral DCA (30 mg/kg) preceding each CCl_4_ challenge (Figure 4A). DCA administration mitigated CCl_4_-induced hepatic injury, as was evident by the significantly lower serum ALT and AST levels observed versus those in the CCl_4_-only controls (Figure 4B). Liver histological analysis demonstrated that DCA-pretreated mice exhibited less severe fibrosis than the untreated fibrotic controls did (Figure 4C). Consistent with this finding, semi-quantitative assessment of fibrotic liver tissues indicated that DCA significantly reduced collagen deposition and the expression of pro-fibrotic mediators, including α-SMA and collagen I (Figure 4D–F). These findings demonstrate that the secondary BA, DCA, has the potential to alleviate CCl_4_-induced hepatic fibrogenesis in mice.

Our preliminary findings indicated increased pro-inflammatory macrophage infiltration in the livers of ABX fibrotic mice. This observation prompted us to investigate whether DCA modulates macrophage polarization in vivo. To this end, hepatic macrophages (F4/80^+^CD11b^+^) were sorted from liver tissue, and CD86^+^ (M1) and CD206^+^ (M2) subsets were further isolated to assess macrophage differentiation states (Figure S2A). Flow cytometry analysis revealed that CCl_4_-induced liver fibrosis not only significantly enhanced total hepatic macrophage infiltration but also markedly skewed macrophage polarization toward the pro-inflammatory M1 phenotype (Figure 4G,H). Although DCA treatment failed to attenuate overall macrophage infiltration, it effectively reversed the fibrosis-driven expansion of M1 macrophages (Figure 4G,H). In contrast, the proportion of M2 macrophages remained comparable between the liver fibrosis and DCA-treated groups (Figure S2B). These results suggest that the anti-fibrotic effect of DCA is mediated, at least in part, by its specific inhibition of M1 macrophage polarization.

### 3.4. DCA Inhibits HSC Activation by Suppressing Macrophage M1 Polarization

**Figure 5:**
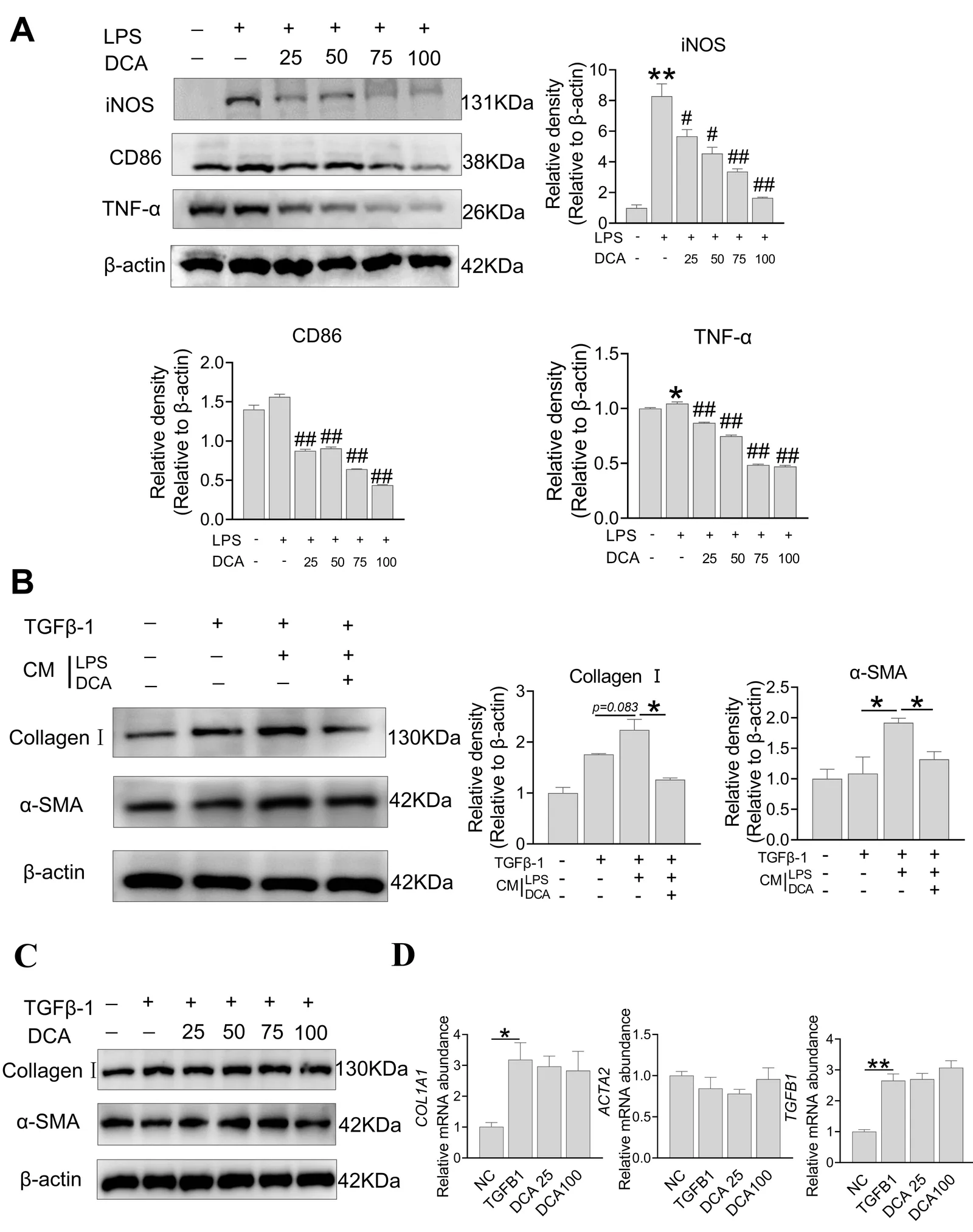
DCA suppresses macrophage M1 polarization to inhibit HSC activation, rather than acting directly on HSCs. (**A**) RAW264.7 cells were treated with the indicated concentrations of DCA for 3 h before being stimulated with LPS for 24 h. Protein levels of M1 macrophage markers were measured. (**B**) LX-2 cells were activated with TGF-β1 (1 ng/mL) and co-cultured with conditioned media (CM) derived from THP-1 macrophages for 24 h. Protein levels of collagen I and α-SMA were detected by Western blotting. (**C**) LX-2 cells were co-treated with TGFβ-1 (10 ng/mL) and varying concentrations of DCA for 24 h. Protein levels of α-SMA and collagen I were determined. (**D**) LX-2 cells were treated with TGFβ-1 (10 ng/mL) alone or combined with DCA (25 μM or 100 μM). The mRNA levels of fibrosis-related genes were determined. Comparisons between two groups were performed using two-tailed Student’s *t*-tests or Mann–Whitney *U* tests, as appropriate. The results are presented as mean ± SEM, n = 3. * *p* < 0.05, ** *p* < 0.01, compared with control; # *p* < 0.05, ## *p* < 0.01 compared with LPS. LPS, lipopolysaccharide; DCA, deoxycholic acid; TGFβ-1/TGFB1, transforming growth factor-β1; NC, normal control; DCA 25, 25 μM of DCA; DCA 100, 100 μM of DCA.

Given the pivotal role that macrophages play in liver inflammation—a key driver of HSC activation [33]—and building on our evidence that macrophages are the primary cellular targets of DCA in ameliorating fibrosis, we subsequently investigated the direct regulatory effect that DCA exerts on macrophage activation in vitro using RAW 264.7 cells and BMDMs. Macrophages were pretreated with DCA (25, 50, 75, 100 μM) for 3 h prior to stimulation with LPS (100 ng/mL) for 6, 12, or 24 h. In RAW 264.7 cells, DCA inhibited the LPS-induced expression of M1 markers (iNOS, CD86, TNF-α) in a dose-dependent manner following 24 h of stimulation (Figure 5A, Figure S3A,B). Similarly, DCA treatment inhibited the LPS-induced upregulation of M1 macrophage markers in BMDMs (Figure S3C).

Having established that DCA directly modulates macrophage polarization in vitro and recognizing the critical crosstalk between macrophages and HSCs in driving liver fibrosis [34], we sought to validate whether DCA indirectly suppresses HSC activation by inhibiting M1 polarization. To this end, we established a THP-1/LX-2 co-culture system. THP-1 macrophages received DCA (50 μM) or a vehicle before LPS stimulation. Conditioned media were then harvested from these cultures. As reported [25] and confirmed in our study, CM from LPS-activated THP-1 macrophages alone failed to induce LX-2 activation (Figure S3D). Therefore, to mimic the pro-fibrotic microenvironment, LX-2 cells were primed with a low dose of TGF-β1 (1 ng/mL). Compared to LX-2 cells treated with TGF-β1 alone (control), co-treatment with CM derived from LPS-activated THP-1 macrophages significantly enhanced LX-2 activation, as was evident by the increased protein levels of collagen I and α-SMA, with the increase in α-SMA reaching statistical significance. Critically, the CM collected from DCA-pretreated and LPS-activated THP-1 macrophages significantly attenuated this pro-fibrotic effect, reversing the expression of these fibrosis markers induced by the pro-inflammatory macrophage CM (Figure 5B).

Considering that HSC activation is a central event in hepatic fibrosis, we then determined whether DCA exerts direct effects on HSCs. LX-2 cells were co-treated with recombinant human TGF-β1 (10 ng/mL) and varying concentrations of DCA for 24 h. HSC activation status was evaluated by quantifying the expression of α-SMA and collagen I at both the mRNA and protein levels. As expected, TGF-β1 stimulation robustly upregulated α-SMA and collagen I expression. However, DCA co-treatment did not significantly affect this TGF-β1-induced upregulation of either α-SMA or collagen I across all concentrations tested (Figure 5C,D). Taken together, these results indicate that DCA ameliorates liver fibrosis primarily by inhibiting pro-inflammatory macrophage activation, which in turn indirectly suppresses HSC activation, with no evidence of a direct effect on HSCs.

### 3.5. DCA Inhibits Macrophage M1 Polarization Through TGR5

**Figure 6:**
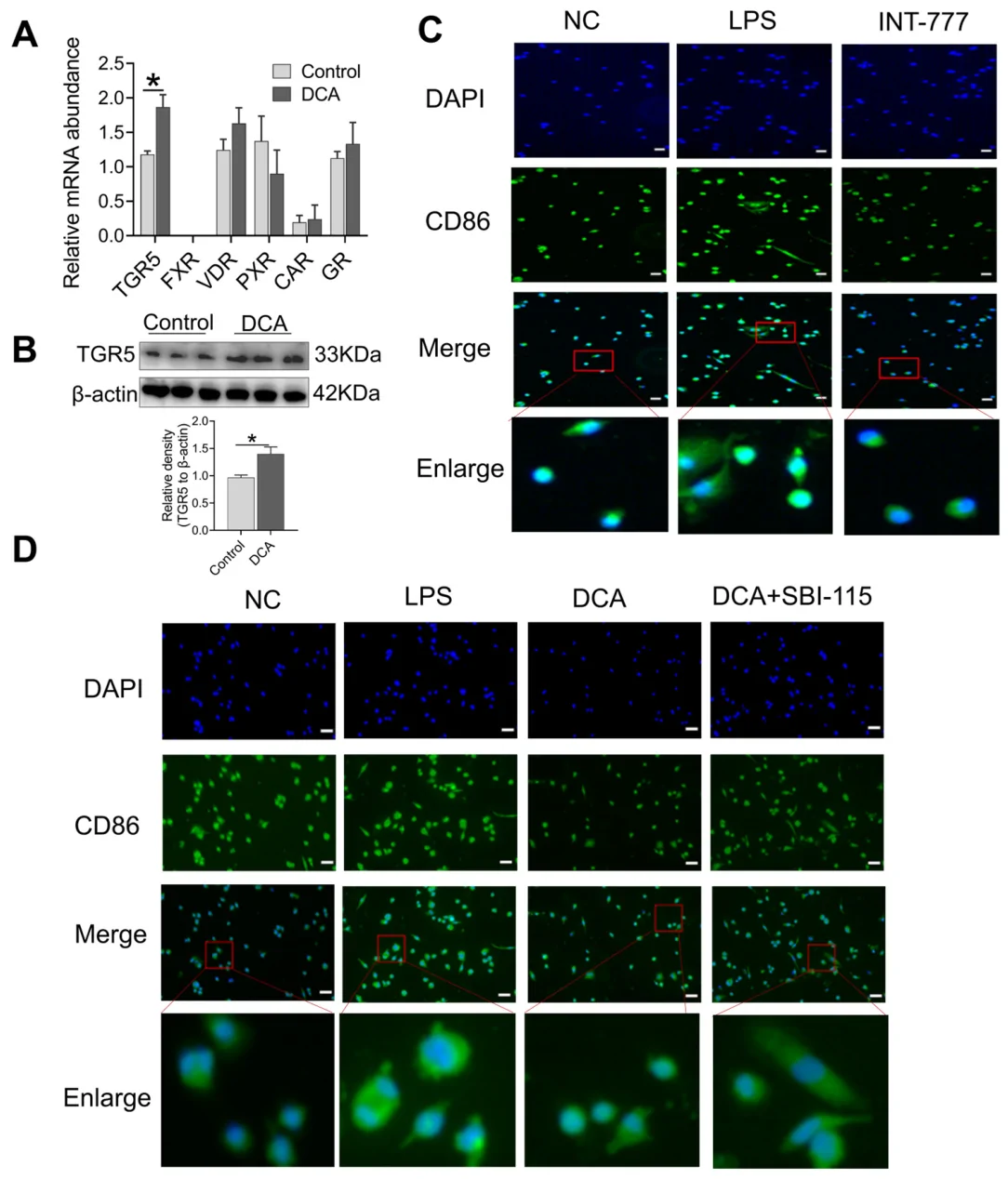
DCA inhibits macrophage M1 polarization through TGR5. (**A**) Expression levels of BA receptors in macrophages treated with DCA. (**B**) TGR5 protein levels in macrophages treated with DCA. (**C**) Representative immunofluorescence staining of CD86 (original magnification ×200, scale bar: 50 μm) in macrophages treated with the TGR5 agonist INT-777. (**D**) Representative immunofluorescence staining of CD86 (original magnification ×200, scale bar: 50 μm) in macrophages treated with LPS, LPS + DCA, or LPS + DCA plus the TGR5 inhibitor SBI-115. Comparisons between two groups were performed using two-tailed Student’s *t*-tests or Mann–Whitney *U* tests, as appropriate. The results are presented as mean ± SEM, n = 3. * *p* < 0.05. NC, normal control; LPS, lipopolysaccharide; DCA, deoxycholic acid.

BAs modulate BA and metabolic homeostasis, as well as immune-inflammatory responses, by activating various receptors, including the farnesoid X receptor (FXR), pregnane X receptor, vitamin D receptor, and TGR5 [35]. Analysis of the mRNA expression of these BA receptors in LPS-stimulated THP-1-derived macrophages revealed that DCA specifically upregulated TGR5 mRNA levels (Figure 6A). Consistently, TGR5 protein expression was markedly increased following DCA treatment (Figure 6B). To investigate whether the inhibitory effect of DCA on macrophage M1 polarization is mediated through TGR5 activation, we used the semi-synthetic TGR5 agonist, INT-777. Immunofluorescence staining demonstrated that INT-777 suppressed the expression of the M1 macrophage marker, CD86, in a concentration-dependent manner (Figure 6C, Figure S4A). A similar inhibitory trend was observed for iNOS protein levels (Figure S4B). Furthermore, the suppressive effect of DCA on M1 polarization was significantly attenuated by treatment with the TGR5 inhibitor, SBI-115 (Figure 6D). Collectively, these findings demonstrate that DCA inhibits macrophage M1 polarization by activating the TGR5 receptor.

### 3.6. TGR5 Activation Suppresses NF-κB Signaling via the cAMP-PKA Pathway in LPS-Stimulated Macrophages

**Figure 7:**
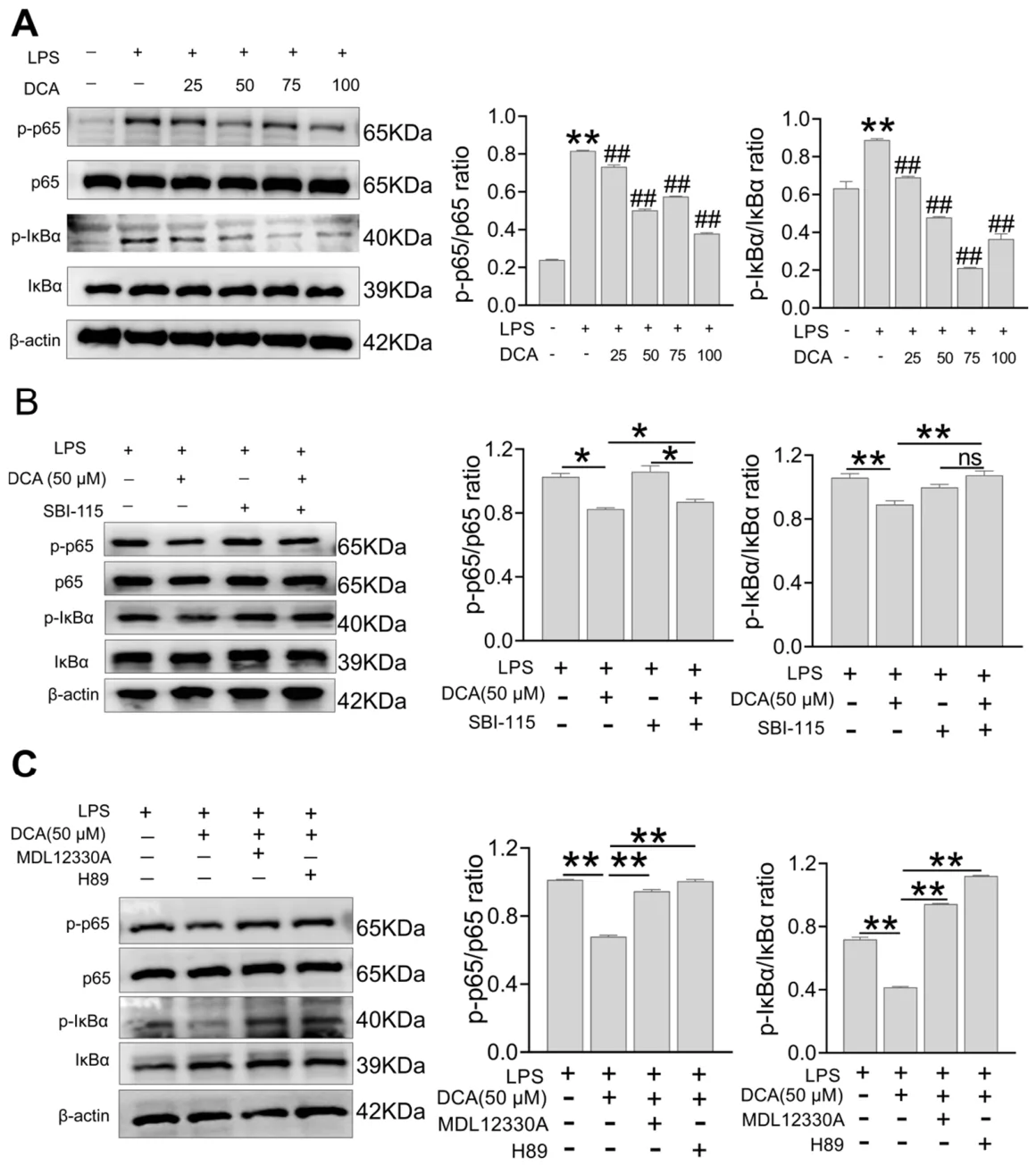
TGR5 activation suppresses NF-κB signaling via the cAMP-PKA pathway in LPS-stimulated macrophages. (**A**) THP-1-derived macrophages were treated with the indicated concentrations of DCA for 3 h before being stimulated with LPS for 1 h. The phosphorylation levels of p65 and IκBα were detected by Western blot. (**B**) THP-1-derived macrophages were treated with LPS, LPS + DCA, LPS + SBI-115, or LPS + DCA plus SBI-115. The phosphorylation levels of p65 and IκBα were detected by Western blot. (**C**) Cells were pretreated with MDL12330A (10 μM) or H89 (10 μM) for 1 h, followed by DCA (100 μM) treatment for 2 h and subsequent LPS stimulation for 1 h. The phosphorylation levels of p65 and IκBα were detected by Western blot. Comparisons between two groups were performed using two-tailed Student’s *t*-tests or Mann–Whitney *U* tests, as appropriate. The results are presented as mean ± SEM, n = 3. * *p* < 0.05, ** *p* < 0.01, compared with control; ## *p* < 0.01 compared with LPS, ns; not significant. LPS, lipopolysaccharide; DCA, deoxycholic acid.

TGR5 exerts anti-inflammatory effects primarily by suppressing the NF-κB signaling pathway [36], where NF-κB is the principal transcription factor governing M1 macrophage polarization [37]. Activation of this pathway is thus critical for M1 macrophage polarization. Our data demonstrated that DCA significantly attenuated the LPS-induced phosphorylation of IκBα and p65 in macrophages (Figure 7A). Moreover, pharmacological inhibition of TGR5 with SBI-115 substantially reversed the DCA-mediated suppression of NF-κB signaling (Figure 7B). Evidence indicates that TGR5 activation modulates downstream signaling via the cAMP–PKA axis [18,38]. To investigate whether the TGR5-dependent inhibition of NF-κB activation is mediated by this cAMP–PKA mechanism, we employed the adenylate cyclase inhibitor, MDL-12330A, and the PKA inhibitor, H89. Pharmacological blockade of adenylate cyclase or PKA abrogated the DCA-dependent suppression of NF-κB signaling (Figure 7C). These findings establish the TGR5–cAMP–PKA–NF-κB cascade as the mechanistic basis for the DCA-mediated suppression of M1 macrophage polarization.

## 4. Discussion

Accumulating evidence has implicated the gut microbiota and its derived metabolites in the progression of liver fibrosis; however, the specific mechanisms involved remain unclear [39,40,41]. Contrasting with this pathogenic role, our study found that microbiota depletion exacerbated liver injury and fibrosis, thereby highlighting the protective role of the commensal microbiota. Notably, we hypothesized that this hepatoprotective effect was mediated by secondary BAs, as their levels were depleted in both microbiota-ablated and fibrotic states. To test this, we supplemented mice with the secondary BA DCA, which significantly attenuated liver fibrosis, thus confirming its beneficial role. At the mechanistic level, DCA activation of the TGR5 receptor inhibited NF-κB-mediated macrophage M1 polarization and subsequent pro-inflammatory signaling, which ultimately suppressed HSC activation and ameliorated fibrosis. Despite interspecies differences, these findings provide mechanistic insights for the development of therapies against clinical liver fibrosis.

Gut dysbiosis is thought to result in a leaky gut, bacterial translocation, and metabolite disorders, ultimately triggering liver inflammation and fibrosis progression [42,43,44,45]. Consistent with this, gut microbiota depletion attenuates acute pancreatitis by reducing bacterial pancreatic translocation [46]. Moreover, antibiotics such as rifaximin have been clinically confirmed to exert preventive and therapeutic effects against hepatic encephalopathy in cirrhotic patients by suppressing the gut microbiota [47,48]. However, whether microbiota depletion improves liver fibrosis remains unclear. Interestingly, and somewhat unexpectedly, ABX-treated mice exhibited more severe liver inflammation and fibrosis than the conventional controls following CCl_4_ challenge, suggesting that bacterial translocation may not be the primary driver of gut dysbiosis in liver fibrotic progression in this context. This aligns with the report of heightened hepatic injury and fibrotic burden in germ-free mice versus conventional mice across multiple fibrosis models [28]. A recent study revealed that gut dysbiosis induces significant microbiota metabolites in cholestatic liver fibrosis and underscores the importance of metabolite intervention for liver fibrosis [49]. Therefore, a more nuanced perspective is needed rather than focusing solely on bacterial translocation, as the role of gut-derived metabolites—such as BAs—may represent a critical mechanistic link between dysbiosis and progressive liver fibrosis.

Gut microbiota-derived metabolites serve as critical mediators of host–microbiota crosstalk and influence diverse pathophysiological processes [50]. Dysbiosis-induced alterations in these metabolites contribute to the progression of liver disease [3]. Our previous study demonstrated that gut dysbiosis in liver fibrosis leads to a definitive decrease in gut microbiota-derived secondary BA levels [29]. This secondary BA deficiency was further exacerbated by experimental gut microbiota depletion, which correlated with aggravated fibrosis. Importantly, accumulating clinical and preclinical evidence has established an inverse relationship between secondary BA levels and the severity of liver fibrosis [15,30,32,51]. In agreement with these results, we found that liver fibrosis was associated with lower levels of secondary BAs, which underwent a further decline after commensal bacterial elimination. We speculated that lower levels of secondary BAs contributed to the worsening of fibrosis. Exogenous supplementation with DCA—the secondary BA most significantly reduced in CCl_4_-induced fibrotic livers—ameliorated liver fibrosis, substantiating the protective effect of specific secondary BAs against fibrosis. Secondary BAs other than DCA, such as lithocholic acid and allo-lithocholic acid, have also been documented to possess hepatoprotective effects against fibrosis [52,53]. Thus, the protective effects of gut commensal bacteria against fibrosis are mediated, at least in part, by specific secondary BAs. The broader class of secondary BAs may harbor important anti-fibrotic functions, which warrants further investigation.

BAs regulate metabolic and inflammatory processes through specific receptors, including nuclear receptors (e.g., FXR) and G protein-coupled receptors (GPCRs) (e.g., TGR5) [54]. While FXR is predominantly expressed in hepatocytes, HSCs, and the intestinal epithelium, TGR5 is selectively overexpressed in innate immune cells—particularly macrophages—where it confers anti-inflammatory protection against hepatic pathologies [35]. Here, we found that DCA inhibited M1 macrophage activation via TGR5. This finding aligns with established reports that TGR5 activation skews macrophages to an M2-functional orientation, suppressing pro-inflammatory responses [38]. Evidence confirms that M1 macrophages actively drive liver fibrogenesis through pro-inflammatory signaling, which directly activates HSCs [55,56]. Consistently, we confirmed that DCA indirectly inhibits HSC activation by inhibiting M1 macrophage polarization rather than by acting directly on HSCs. Collectively, we established that DCA-mediated TGR5 activation in macrophages disrupts pro-fibrotic inflammatory signaling, representing a key mechanism for BA-dependent liver protection.

As with all GPCRs, TGR5 activation stimulates adenylyl cyclase, elevating cAMP levels and activating PKA, thereby initiating downstream signaling cascades [57]. TGR5 activation induces PKA-dependent NLRP3 phosphorylation at Ser291 in macrophages. This modification promotes NLRP3 ubiquitination, thereby suppressing NLRP3 inflammasome activation [38]. Furthermore, the TGR5–cAMP–PKA axis inhibits IκBα phosphorylation across multiple cell types, consequently blocking NF-κB activation [18,27,58]. Consistent with these mechanisms, our study demonstrated that DCA inhibits M1 macrophage polarization and exerts anti-inflammatory effects. This occurs through the TGR5–cAMP–PKA signaling cascade, which suppresses the phosphorylation of IκBα and p65 within the NF-κB pathway. Given that NF-κB is a well-established central mediator in hepatic injury, fibrosis, and HCC [59], targeting this pathway via BAs and TGR5 holds significant therapeutic potential for liver injury and fibrosis.

In conclusion, we established that the gut microbiota confers protection against hepatic inflammation and fibrosis, at least in part, via secondary BA metabolism. Mechanistically, microbiota-derived secondary BAs suppress NF-κB-driven M1 macrophage polarization by activating the TGR5–cAMP–PKA axis, thereby attenuating inflammation and fibrosis. Despite these insights, three limitations of this study warrant consideration. First, while focusing on BA metabolism limits extrapolation to other microbial metabolites, their potential contribution to fibrotic progression cannot be excluded. Second, the role of TGR5 signaling in secondary BA-mediated protection requires verification using macrophage-specific knockout models. Third, the putative protective role of microbiota-generated secondary BAs in patients with CLD requires rigorous validation. Notwithstanding these constraints, our study delineates the gut microbiota–BA axis as a key regulator of hepatic fibrosis, unveiling TGR5-mediated macrophage reprogramming as a druggable target for anti-fibrotic therapy development.

## Data Availability

The data of 16S rRNA gene sequencing have been deposited in the SRA database in NCBI (PRJNA1302345, https://dataview.ncbi.nlm.nih.gov/object/PRJNA1302345?reviewer=75peqvi4j32javjhrt45afk762, accessed on 12 August 2026) and are publicly available as of the date of publication. Any additional information required to reanalyze the data reported in this paper is available from the corresponding author, zhangjie_cq@cqu.edu.cn, upon reasonable request.

## Supplementary Materials

The following supporting information can be downloaded at https://www.mdpi.com/article/10.3390/microorganisms14092035/s1, Figure S1. ABX treatment depletes the gut microbiota and reduces its diversity in mice. Figure S2. DCA regulates macrophage polarization in vivo. Figure S3. DCA inhibits macrophage polarization towards the M1 phenotype in vitro. Figure S4. TGR5 activation inhibits macrophage M1 polarization. Table S1. Primer sequences for RT-PCR.

## Abbreviations

The following abbreviations are used in this manuscript:

ABX	Antibiotic cocktail
ALT	Serum alanine aminotransferase
AST	aspartate aminotransferase
ASV(s)	Amplicon sequence variant(s)
BA(s)	Bile acid(s)
CCl_4_	Carbon tetrachloride
CLD(s)	chronic liver disease(s)
CM	Conditioned media
DCA	Deoxycholic acid
DMEM	Dulbecco’s modified Eagle medium
ECM	Extracellular matrix
FBS	Fetal bovine serum
FXR	Farnesoid X receptor
HCC	Hepatocellular carcinoma
HSC	Hepatic stellate cell
LPS	Lipopolysaccharide
M-CSF	Recombinant mouse
RPMI	Roswell Park Memorial Institute
SR	Sirius Red
TGF-β1	Transforming growth factor beta
TGR5	Takeda G protein-coupled receptor 5
α-SMA	Alpha-smooth muscle actin
