# Supplementary material for: Gut Commensal Bacteria Ameliorate Liver Fibrosis by Inhibiting Macrophage M1 Polarization Through Secondary Bile Acids in a Murine Liver Fibrosis Model
Source: Microorganisms. 2026 Sep 12;14(9):2035. doi: 10.3390/microorganisms14092035 (PMC13609333; doi:10.3390/microorganisms14092035)
Supplement: Supplementary file 1 [file microorganisms-14-02035-s001.zip › microorganisms-4515217-supplementary.pdf]

Supplementary materials

Figure S1

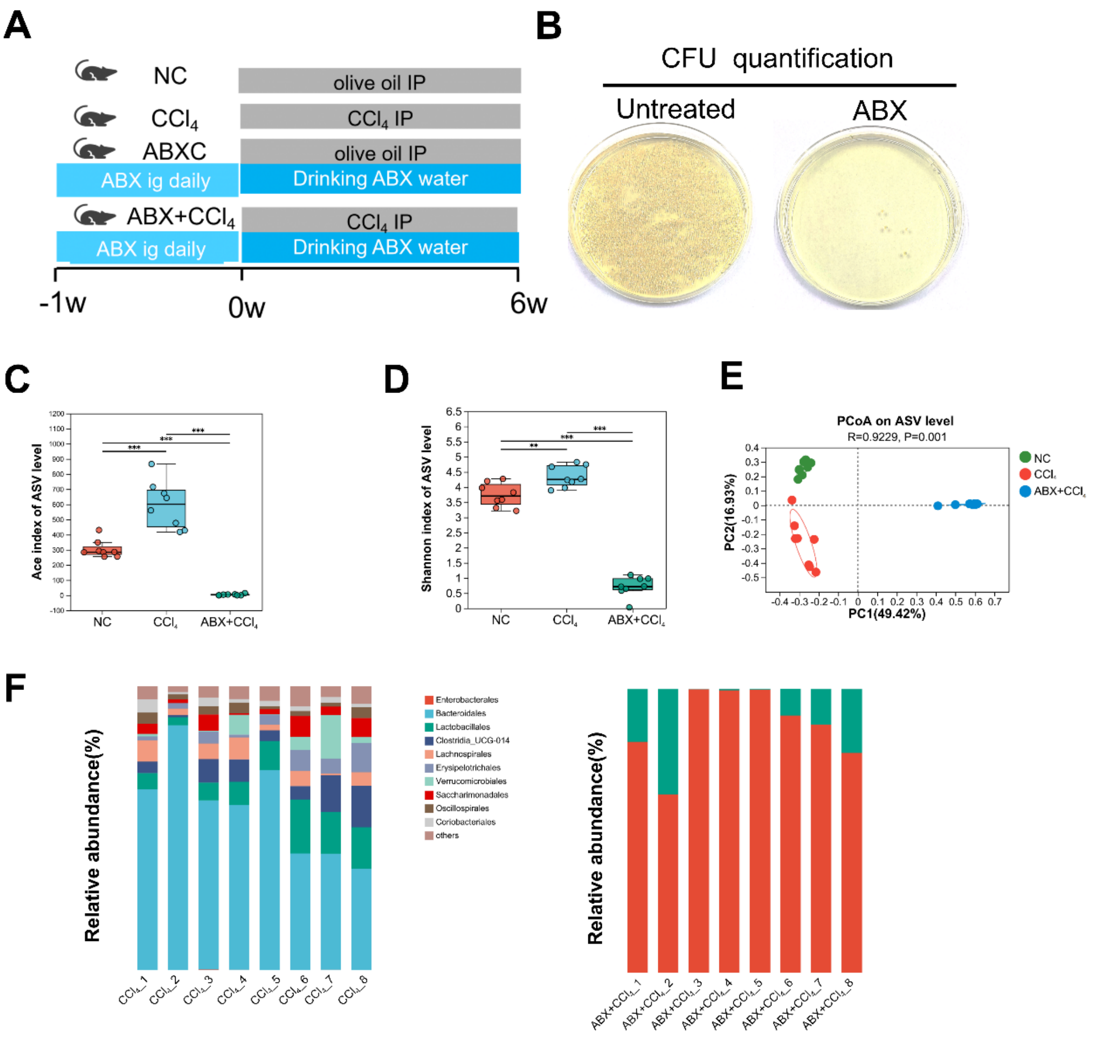

Figure S1. ABX treatment depletes the gut microbiota and reduces its diversity in mice. (A) Experimental schematic. (B) Colony-forming unit (CFU) quantification from cecal contents cultured aerobically (±ABX treatment). (C-D) ABX treatment caused near-complete loss of microbiota alpha diversity. (E) Principal coordinates analysis (PCoA) plots based on Bray-Curtis dissimilarity, showing distinct clustering of fecal microbiota communities among normal control mice, CCl_4-induced liver fibrosis mice, and ABX-treated liver fibrosis mice. (F) Relative abundance of fecal microbiota at the order level in control and ABX-treated liver fibrosis mice. *$p < 0.05$, **$p < 0.01$, *** $p < 0.001$. NC, normal control; CCl_4, carbon tetrachloride; ABX, broad-spectrum antibiotic cocktail; ABXC, ABX control.

Figure S2

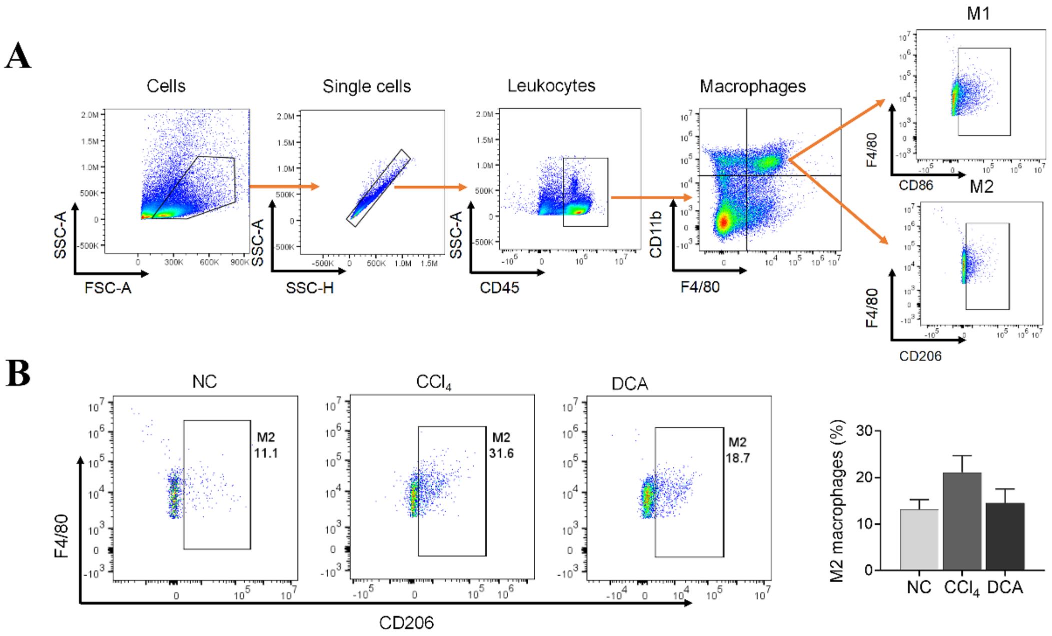

Figure S2. DCA regulates macrophage polarization in vivo. (A) Gating strategy for macrophages in FCM. (B) Representative FCM plots of hepatic M2 macrophages.

Figure S3

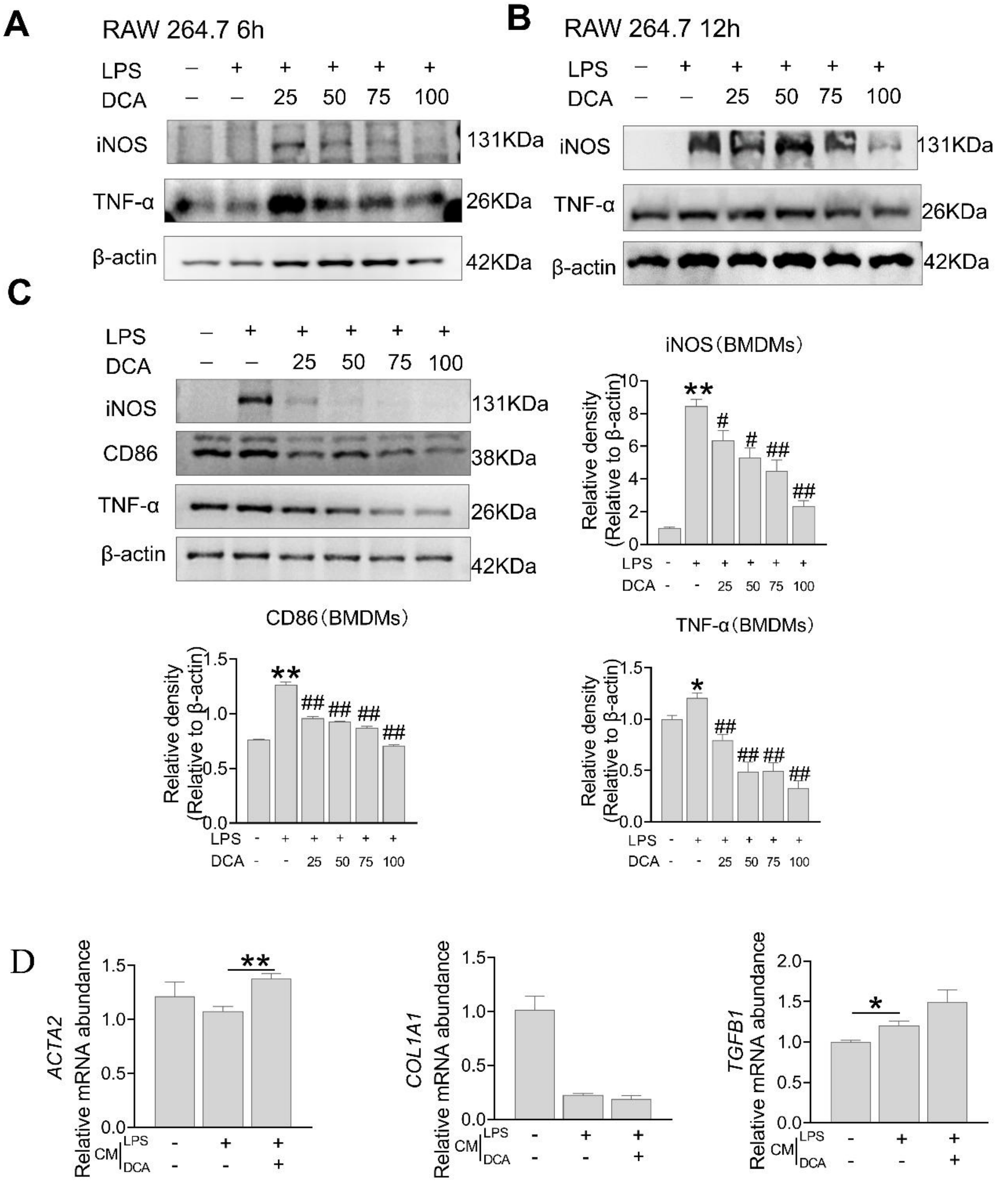

Figure S3. DCA inhibits macrophage polarization towards the M1 phenotype in vitro. (A-B) RAW 264.7 cells were pretreated with the indicated concentrations of DCA for 3 h prior to stimulation with LPS for 6 h or 12 h. Protein expression levels of M1 macrophage-related markers were measured. (C) Bone marrow-derived macrophages (BMDMs) were pretreated with the indicated concentrations of DCA for 3 h prior to stimulation with LPS for 24 h. Protein expression levels of M1 macrophage-related markers were measured. (D) LX-2 cells were co-cultured with

conditioned medium (CM) derived from THP-1 macrophages for 24 h. Protein levels of α-SMA and collagen I were measured by western blotting. *$p < 0.05$, **$p < 0.01$, compared with control; #$p < 0.05$, ##$p < 0.01$ compared with LPS. LPS, lipopolysaccharide; DCA, deoxycholic acid; TGFβ-1/TGFB1, transforming growth factor-β1.

Figure S4

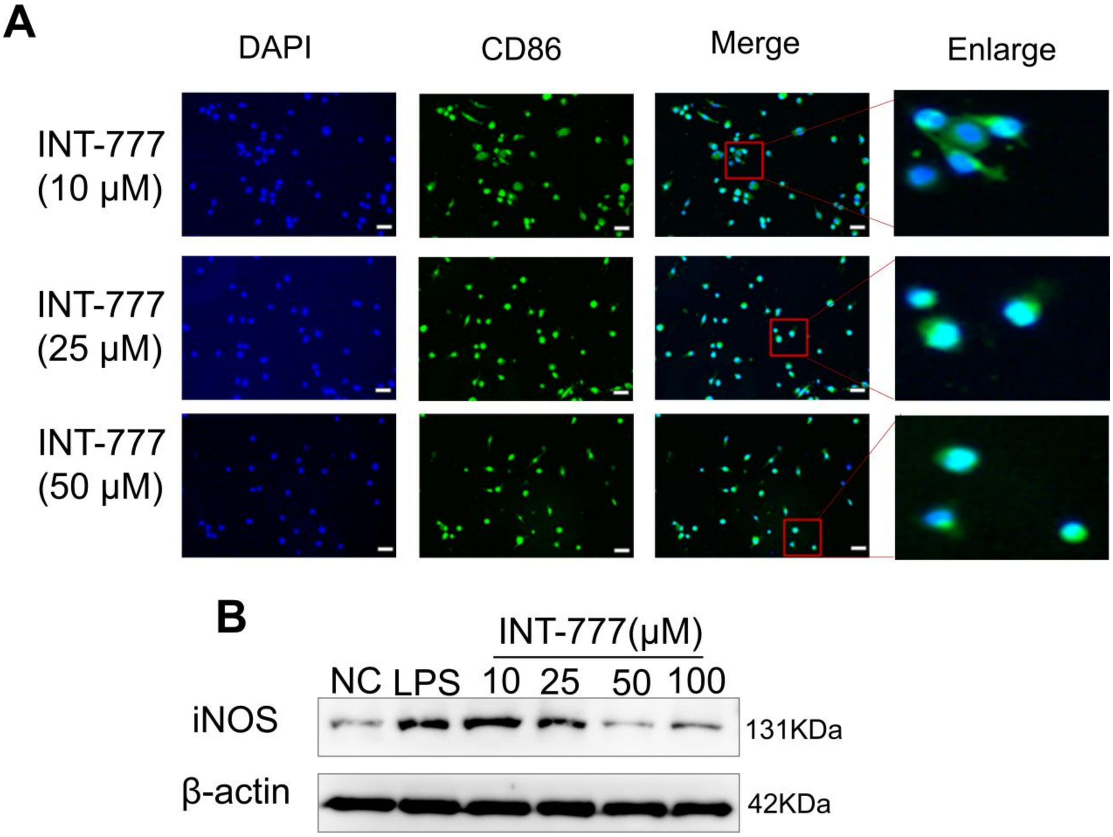

Figure S4. TGR5 activation inhibits macrophage M1 polarization. (A) Immunofluorescence staining of CD86 (×200 magnification, scale bar: 50 μm) in macrophages treated with indicated concentrations of INT-777. (B) Protein levels of iNOS in THP-1-derived macrophages pretreated with indicated concentrations of INT-777 for 3 h and stimulated with LPS for 24 h. NC, normal control; LPS, lipopolysaccharide.

Table S1. Primer sequences for RT-PCR.

gene name	direction	5' to 3'
Mouse *Cd45*	Forward	GCACTCGGGCTTTCAAAGATATT
	Reverse	AAGCATTAACTCTCCGTCGAAGT
Mouse *Mcp1*	Forward	GGTGTCCCAAAGAAGCTGTAGTT
	Reverse	AGCTTCAGATTTACGGGTCAACT
Mouse *F4/80*	Forward	TGGTGGTCATAATCTCTGCTTCT
	Reverse	CAGGCAAGGAGGACAGAGTTTAT
Mouse *Cd86*	Forward	CATGGGCTTGGCAATCCTTATCT
	Reverse	TTGTAAATGGGCACGGCAGATAT
Mouse *Nos2*	Forward	GTCCGAAGCAAACATCACATTCA
	Reverse	GGTAAACACGTTCTTTGCATGGA
Mouse *Il6*	Forward	AACCGCTATGAAGTTCCTCTCTG
	Reverse	TGGTATCCTCTGTGAAGTCTCCT
Mouse *Il1b*	Forward	CAGCACATCAACAAGAGCTTCAG
	Reverse	GAGGATGGGCTCTTCTTCAAAGA
Mouse *Tnf*	Forward	GCCTCCCTCTCATCAGTTCTATG
	Reverse	ACCTGGGAGTAGACAAGGTACAA
Mouse *Tgfb1*	Forward	GGAAATCAACGGGATCAGCC
	Reverse	AGTTGGTATCCAGGGCTCTC
Mouse *Col1a1*	Forward	AGCACGTCTGGTTTGGAGAG
	Reverse	GACATTAGGCGCAGGAAGGT
Mouse *Acta2*	Forward	GTACCACCATGTACCCAGGC
	Reverse	GCTGGAAGGTAGACAGCGAA
Mouse *Gapdh*	Forward	GTGTGAACGGATTTGGCCGT
	Reverse	TGCCGTTGAATTTGCCGTGA
Human *TGFB1*	Forward	TACAGCAACAATTCCTGGCGATA
	Reverse	CAGTGTGTTATCCCTGCTGTCAC
Human *COL1A1*	Forward	CCCAGCCACAAAGAGTCTACAT
	Reverse	GATTGGTGGGATGTCTTCGTCTT
Human *ACTA2*	Forward	CCTGACCCTGAAGTACCCGATAG
	Reverse	GGCAACACGAAGCTCATTGTAGA
Human *TGR5*	Forward	TCGACCTGGACTTGAACTAAAGG
	Reverse	TAAACGGCCAGAGGAGCTTTATT
Human *FXR*	Forward	GGGCAACTGTGTGATGGATATGT
	Reverse	GTCACGACCTTCACTGTCTTCAT
Human *VDR*	Forward	ACTGGCTTTCACTTCAATGCTATG
	Reverse	TGCACTTCCTCATCTGTCAGAAT
Human *PXR*	Forward	TTCGAGCTGTGTCAACTGAGATT
	Reverse	CATCAGCACATACTCCTCCTCAT

Human *CAR*	Forward	ACTGAGTAAGGAGCAAGAAGAGC
	Reverse	AAAGTGTTGATGTCTGCGAAGTG
Human *GR*	Forward	TGGTGTCACTGTTGGAGGTTATT
	Reverse	TGCAGTAGGGTCATTTGGTCATC
Human *GAPDH*	Forward	GGAGTCCACTGGCGTCTTCA
	Reverse	GTCATGAGTCCTTCCACGATACC
